# A Highly Efficient HMI Algorithm for Controlling a Multi-Degree-of-Freedom Prosthetic Hand Using Sonomyography

**DOI:** 10.3390/s25133968

**Published:** 2025-06-26

**Authors:** Vaheh Nazari, Yong-Ping Zheng

**Affiliations:** 1 Department of Biomedical Engineering, The Hong Kong Polytechnic University, Hong Kong 999077, China; v.nazari@uq.edu.au; 2 Research Institute for Smart Ageing, The Hong Kong Polytechnic University, Hong Kong 999077, China

**Keywords:** advanced prosthetics, artificial intelligence, prosthetic, human–machine interface, real-time controlling system, sonomyography, wireless ultrasound

## Abstract

Sonomyography (SMG) is a method of controlling upper-limb prostheses through an innovative human–machine interface by monitoring forearm muscle activity through ultrasonic imaging. Over the past two decades, SMG has shown promise, achieving over 90% accuracy in classifying hand gestures when combined with artificial intelligence, making it a viable alternative to electromyography (EMG). However, up to now, there are few reports of a system integrating SMG together with a prosthesis for testing on amputee subjects to demonstrate its capability in relation to daily activities. In this study, we developed a highly efficient human–machine interface algorithm for controlling a prosthetic hand with 6-DOF using a wireless and wearable ultrasound imaging probe. We first evaluated the accuracy of our model in classifying nine different hand gestures to determine its reliability and precision. The results from the offline study, which included ten healthy participants, indicated that nine different hand gestures could be classified with a success rate of 100%. Additionally, the developed controlling system was tested in real-time experiments on two amputees, using a variety of hand function test kits. The results from the hand function tests confirmed that the prosthesis, controlled by the SMG system, could assist amputees in performing a variety of hand movements needed in daily activities.

## 1. Introduction

Hands help to perform the majority of human activities in daily living, and losing one or both hands results in independence reduction [1]. Even though most artificial limbs used today are either purely cosmetic or serve a practical purpose with limited functionalities (such as a hook-like gripper), various multi-fingered prosthetic hands have been developed and commercialized [2,3,4], including the i-Limb Hand, KIT hand, Michelangelo Hand, Bebionic Hand, and Vincent Hand, all of which depend on electrical motors and complex mechanical components. Moreover, the invention of additive technology revolutionized manufacturing methods by decreasing the cost of production and the weight of robots, as well as speeding up the product development process. This invention also affects the industry of prostheses, encouraging researchers and engineers to create numerous 3D-printed prosthetic hands [5,6,7,8,9,10].

Despite the advancements in developing novel, dexterous, and state-of-the-art prosthetic hands with the ability to assist amputees in performing different daily activities [11,12,13], around 50–70% of patients refuse to wear and use current prosthetic hands due to their poor functionality, high cost [8,14,15,16,17,18], low comfort, lack of sensory feedback, and most importantly, inaccurate controlling system, not being able to effectively predict users’ intended movements and provide natural-like control over prostheses [11,19].

To identify the most important features of upper-limb prostheses, several studies have been conducted. The key factors can be listed as anthropomorphic characteristics (kinematics, size, weight, and appearance) [3,20], performance (speed, force, and dexterity) [21,22,23], and strong and integrative grasping [14,24,25]. Bioinspired motion speeds and adequate grip force are necessary for the device to be useful for carrying out the activities of daily living (ADLs) [26]. However, among the most fundamental needs for a robotic prosthesis is the capability to control the robot with sufficient precision and responsiveness of the fingers so that it may be used effectively and with sufficient dexterity [14,27,28,29].

Despite the study of various human–machine interfaces (HMIs), there is still a lack of prosthetics with the reliable control of multiple degrees of freedom [11]. For instance, using biological signals such as electromyography (EMG) or electroencephalography (EEG) as non-invasive approaches have been studied and proposed as popular HMIs, enabling users to control rehabilitation devices not only for prostheses but also rehabilitation robots [30] and exoskeletons [31,32]. However, these techniques are very noisy, and the recorded signals can be affected by electrode movements as well as sweating [33]. Also, EMG sensors are not able to monitor deep muscle activities, making this controlling system unable to be used in predicting more complex hand gestures with acceptable accuracy. For EEG control, the response time is still relatively slow [34,35,36]. In addition, the intended hand gestures performed by robots are limited, and the most commercialized EMG-controlled prostheses still only have close and open functions, although different approaches for controlling robots with high dexterity have been proposed at the research level. 

In recent years, in order to improve the quality of signals recorded from sensors as well as decrease the amount of noise, invasive techniques such as implanted EMG, targeted muscle reinnervation, myoelectric implantable recording arrays (MIRAs) [37], magnetomicrometry (MM) [38], and others have been proposed. However, invasive approaches raise numerous questions regarding safety and efficacy since the electrodes need to be implanted into the body [33]. The field has been searching for a signal which can represent individual muscle activation and be collected non-invasively.

Over the last two decades, using signals extracted from the ultrasound images of muscle during contraction to control prosthetic hands has been a popular research topic. Zheng et al. first studied the feasibility of controlling robotic hands using an ultrasound device in 2006, in which the term “sonomyography” (SMG) was proposed by the team for this non-invasive HMI approach [39]. Basically, SMG refers to the signal representing architectural changes in a muscle detected via real-time ultrasound images during its contraction [40]. Since ultrasound imaging can inherently differentiate the activities of both deep and superficial muscles as well as a group of neighboring muscles simultaneously and non-invasively, SMG has attracted the attention of a lot of researchers since it was proposed [41,42,43,44,45,46,47]. Recently, a unique mobile SMG system to monitor muscles’ activities was evaluated regarding its reliability and validity by Ma et al. in 2019, paving the way for the real-time monitoring of muscle activity throughout both indoor and outdoor activities, especially for controlling prostheses using a wireless SMG system [48].

A number of SMG-based prosthesis control systems have already been reported in the literature, which mainly focus on the demonstration of feasibility, including using single-element transducers [49]. A low-power SMG system designed for wearable use with a prosthetic hand was proposed by Engdahl et al. in 2020 [50]. Using AI to classify intended hand gestures, the authors demonstrated that their suggested technique successfully classified nine distinct finger motions with an accuracy of around 95%. In 2020, Yang et al. [51] advocated for the use of wearable 1D SMG (A-mode ultrasound transducer) equipment in combination with subclass discriminant analysis (SDA) and principal component analysis (PCA) to predict wrist rotation (pronation/supination) and finger movements. This research demonstrated that the SDA machine learning method could be used to identify both finger gesture and wrist rotation concurrently with accuracies of around 99.89% and 95.2%, respectively.

To overcome the difficulties caused by single-element transducers, a number of studies reported the use of B-mode imaging transducers [49]. In a study published in 2019, Akhlaghi et al. [52] evaluated the effect of using a sparse set of ultrasound scanlines to determine the optimal location on the forearm for capturing the maximal deformation of the primary forearm muscles during finger motions and classifying various types of hand gestures and finger movements. The results indicated that the ultrasonic probe should be placed over around 40–50% of the forearm’s length in order to identify distinct hand motions with greater precision. This is because the largest muscle activation occurs in this region. In addition, the categorization result demonstrated that employing B-mode ultrasound to operate a prosthetic hand was a viable option, since the accuracy was almost 95%. In 2019, Li et al. [53] tested the capabilities of M-mode and B-mode ultrasound to detect 13 various hand and finger movements in eight able-bodied subjects. Using the Support Vector Machine (SVM) algorithm to classify various hand gestures, the accuracy of the M-mode classification was determined to be 98.83 ± 1.03%, and that of the B-mode classification was determined to be 98.77 ± 1.02%. On the other hand, the accuracy of the Backpropagation Artificial Neural Network (BP-ANN) classifier was 98.77% in M-mode and 98.76% in B-mode. They discovered that M-mode SMG transducers were equally as accurate as B-mode SMG signals when it came to detecting wrist and finger movements, as well as in differentiating between a variety of hand gestures, which suggests their possible utility in human–machine interfaces.

Zheng et al. in 2006 [39] and Guo et al. in 2008 [54] conducted the first experiments to evaluate the relationship between morphological changes in forearm muscles and wrist angle. The results of their studies showed that muscle deformation measured by ultrasound correlated linearly with wrist angle. Moreover, in 2011 and 2012, Castellini et al. [55,56] conducted exciting experiments to assess the potential of an SMG system in predicting the position of the fingers using ultrasound images collected from human forearms. The results of their studies, by discovering a linear relationship between finger position and the extracted features from ultrasound images, showed that this novel controlling system had great potential not only for predicting intended hand gestures, but also for providing information regarding finger position and the amount of flexion, enabling the SMG controlling system to provide a proportional and natural-like control experience to people with amputations.

For a more complete understanding of the various systems and techniques developed using the ultrasound imaging of muscle, or SMG, for controlling upper-limb prostheses, readers can refer to a review paper recently published by Vaheh et al. in 2023 [57], which conducted a comprehensive evaluation and comparison of the results and findings of previously published works on SMG systems as novel human–machine interfaces. The outcomes of this review paper demonstrated the promise of ultrasonic sensing as a practical human–machine interface for the control of bionic hands with multiple degrees of freedom. In addition, this review showed that a variety of machine learning algorithms combined with feature extraction models could correctly classify various hand gestures with an accuracy of about 95% [57].

Building on these insights, and considering other mainstream technologies including EMG, we prepared a comparison table (Table S1) that summarized the key factors—accuracy, latency, weight, and cost—across these leading technologies used in prosthetic hand control [57,58,59,60,61,62,63,64,65].

Despite all the feasibilities demonstrated about using SMG together with machine learning or deep learning methods for detecting hand gestures with the potential for prothesis control, there are few reports about testing an SMG-control-based robot on real amputees [66]. Considering that residual muscles after amputation surgery are very different from those in normal subjects, the promising results demonstrated in earlier papers may not necessarily stand with residual limbs. In addition, up to now, there is still no report of a system integrating SMG together with a prothesis for testing on amputee subjects to demonstrate its performance in daily activities.

**Figure 1:**
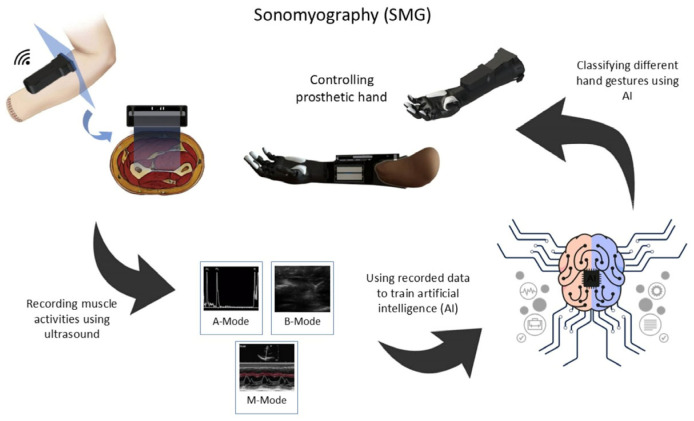
Sonomyography as a novel HMI method: The overall schematic of the sonomyography (SMG) setup and control of the prosthetic hand using an ultrasound probe.

In this study, we report the design and performance of a novel SMG controlling system, a lightweight (360 g), functional, and cost-effective 6-DOF prosthetic hand called ProRuka (Figure 1). The novel ProRuka was developed and tested by considering anthropomorphism, functionality, safety, and comfort, all of which were inspired by the structure of the human hand. To evaluate the accuracy of the proposed machine learning model in classifying the different hand gestures needed in daily activities, ten able-bodied volunteers were recruited to attend our first experiment. For the offline evaluation experiment, data were first collected from the ten able-bodied participants before being used to train the model and assess the accuracy of the AI model. Among all the data collected from the ten volunteers, around 70% of it was used for training and the rest for validation. For the amputee subjects, data were collected from the individual residual limbs and used to train their individual models. This step is very similar to the training session for using conventional EMG-controlled protheses. The trained model, together with the prosthesis and the controlling system, was evaluated with two amputee subjects, who performed standardized hand function tests including the Box and Blocks (B&B) test, Targeted Box and Blocks (TB&B) test, and Action Research Arm Test (ARAT).

## 2. System and AI Model Development

### 2.1. Programming Environment and Tools

In this study, the primary programming language used was Python 3.10. The main libraries utilized in our code included TensorFlow for deep learning and NumPy for numerical computations. Additionally, the scikit-learn (sklearn) library was employed for implementing various machine learning algorithms and model evaluation. All the key parameters for the models have been specified to facilitate the replication of the results.

### 2.2. Classification of Different Hand Gestures Using Ultrasound Imaging

For the control part reported in this paper, different classification methods were studied. The participants were divided into able-bodied and amputee groups. Each volunteer was asked to sit in a comfortable position and put their hand on a cushion. Then, the muscle activities in different hand gestures were captured using a palm-sized wireless ultrasound probe. The collected images were first resized into 32 × 32 pixels (from 912 × 912 pixels) and then normalized from a [0, 255] to [0, 1] pixel intensity. After that, a convolutional neural network (CNN) was used to extract the features from each image, and these features were used to train a model with a machine learning or regression algorithm. We used Random Forest (RF) with 100 estimators and the random state of 100, k-nearest neighbors (KNN) with 10 neighbors, SVM, and a Decision Tree Classifier (DTC) with a maximum depth of 10 and a minimum sample split of 2 as the machine learning algorithms to train the model, while to train the model using a regression algorithm, we used decision tree regression (max_depth = 3), nearest neighbor regression (n_neighbour = 5), and support vector regression (C = 100, gamma = 0.1, epsilon = 0.1) with two different kernels, linear (SVR-L) and polynomial (SVR-P). Then, the accuracy of each machine learning algorithm was examined and compared.

#### 2.2.1. Feature Extraction

Since the machine learning algorithms could not process all the raw information contained in the images, a CNN algorithm with pretrained weights was used to extract the features from the collected data. Then, these extracted features were utilized to train the AI model. Three different pretrained models including VGG16, VGG19, and InceptionResNetV2 were individually used for feature extraction. To select and extract features, 64 filters from the first convolutional layer were utilized. The features extracted from the training data were then used for classification. It is important to note that using more filters increases the number of extracted features, which can increase the accuracy of AI models. However, more time and GPU memory are required to train models with more extracted features.

#### 2.2.2. Classification

**Figure 2:**
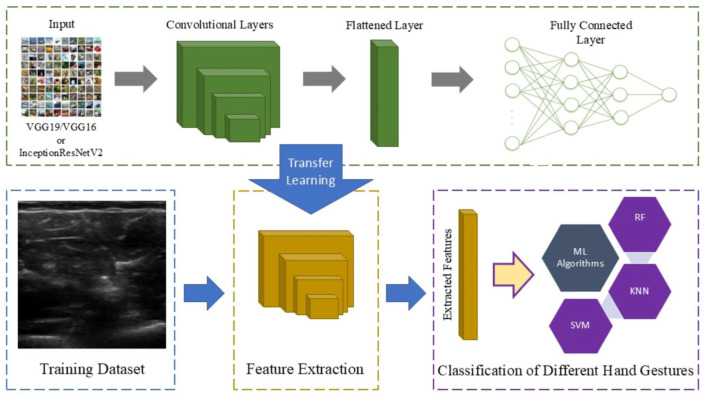
The overall schematic of the classification process: A transfer learning model was used to extract features from the images and the extracted features were then utilized to train the model using machine learning algorithms.

Figure 2 shows the overall schematic of the whole classification process. After extracting the features, these data were used for training three different machine learning classification algorithms, including RF, KNN, DTC, and SVM, as well as four regression algorithms (decision tree regression, nearest neighbor regression, SVR-L, SVR-P) to classify different hand gestures and finger movements. Two-thirds of the collected data were used for training and the rest were applied for validation.

### 2.3. Replacing Ultrasound Gel and Gel Pad with Sticky Silicone Pad

**Figure 3:**
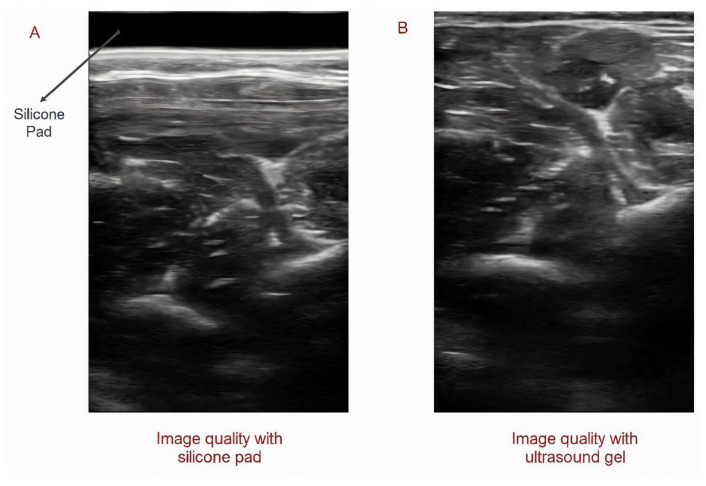
Utilizing silicone pad instead of ultrasound gel: Image quality using silicone pad (**A**) and ultrasound gel (**B**).

For the sticky silicone pad, biocompatible silicone liquid (Deping, Guangdong Province, China) was used to create a pad using the molding technique. In the experiment, two different silicones with hardness ratings of Shore 00-00 and Shore 00-05 were utilized. Three different silicone pads were created for the experiment. The first one was a silicone pad with a hardness of 0. The ultrasound images had a good resolution using this pad, but it was too sticky, and it was difficult to put it on the hand with the prosthesis. A second silicone pad with a hardness of 05 was created. The resolution was good for controlling the prosthesis, but the pad was fragile and could be damaged easily during application and removal. Thus, for the third pad, a combination of silicones with 00 and 05 hardness were mixed together in a 3:1 ratio. The testing results demonstrated that the image quality provided by this silicone gel pad was good enough for controlling the robot, and it was sticky enough to minimize transducer movement. Additionally, the flexibility of the pad was good enough to be used with a socket without any damage (Figure 3). 

### 2.4. Designing a Novel Prosthetic Hand

**Figure 4:**
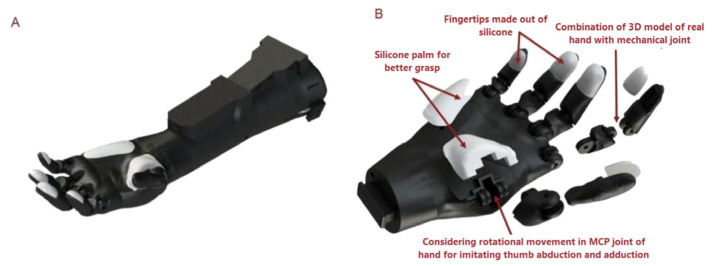
The design of ProRuka: (**A**) The design of ProRuka, a 3D-printed, lightweight, cost-effective, and multi-degree-of-freedom prosthetic hand; (**B**) an exploded view of the prosthesis.

To evaluate the novel SMG controlling system in this study, we developed a low-cost, lightweight, user-friendly, dexterous, multiple-degree-of-freedom, functional prosthetic hand (Figure 4A). To make the prosthetic hand resemble a normal human hand, a 3D model of a normal human hand was first prepared using a portable industrial 3D scanner (EinScan Pro 2x, Shining 3D, Hangzhou, Zhejiang, China). Then, the hand joints of the scanned model were replaced with mechanical joints to make the prosthesis functional (Figure 4B). Moreover, by considering the important role of the abduction and adduction of the thumb in grasping different types of objects and performing 80% of daily living hand activities, we considered the rotational movement in the MCP joint in the design of our prosthetic hand (Figure 4B). In addition, to increase the friction between objects and the prosthesis and decrease the chance of objects slipping and falling from the prosthesis, the fingertip of each finger as well as the palm of prosthesis was made of silicone (Platinum Cure Silicone Rubber Compound with a shore hardness of 00-50, Smooth-On, Macungie, PA, USA). Furthermore, 3D printing technology with black nylon material was utilized (VPrint 3D, Hong Kong, China) to make the prosthesis cost-effective and lightweight.

## 3. Experiment and Results

### 3.1. Participants

Since musculoskeletal anatomy is different between able-bodied people and those with transradial limb loss, it was important to assess the accuracy of the proposed classification method for both groups. Consequently, we separated the participants into able-bodied and amputee groups. The study was approved by the Human Subjects Ethics Sub-committee of The Hong Kong Polytechnic University (HSEARS20220720001).

Ten able-bodied volunteers (five males and five females, aged between 22 and 33) were recruited for experiment 1 (the healthy group), and two amputee subjects (both males aged between 45 and 69, respectively, referred to as A1 and A2) were recruited for experiment 2 (the amputee group). Both amputee subjects had undergone left-hand transradial amputation, 26 and 45 years after their injury, respectively. Each participant completed an informed consent form after receiving information about the research and the experimental design.

### 3.2. Experimental Setup

**Figure 5:**
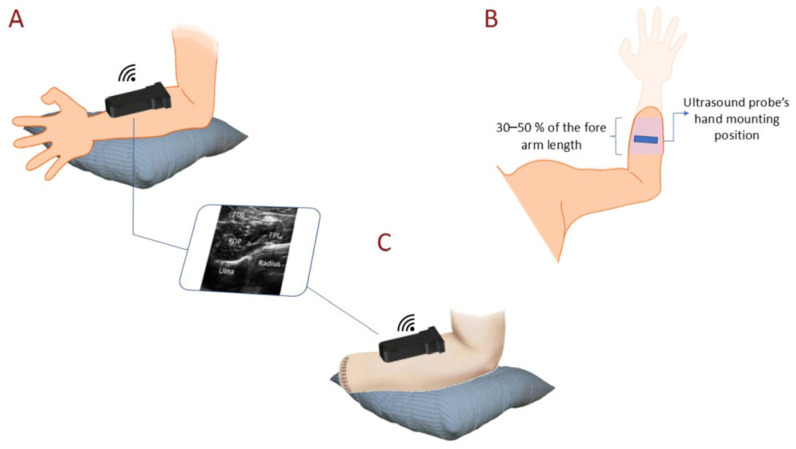
The experimental setup: (**A**) The setup for data collection to test the performance of offline classification (experiment 1) and collect ultrasound images of the main muscles responsible for finger flexion. (**B**) The area on the forearm used to capture the best muscle activities to control the robot. (**C**) The data collection setup used to train the model for functional testing (experiment 2).

The volunteers were asked individually to sit in a comfortable position, put their hand on a cushion, and keep their palm upwards. A B-mode lightweight (only 67 g) wireless ultrasound module (Model UL-1C, Beijing SonopTek Limited, Beijing, China) was fixed on the forearm using a customized case (Figure 5A,B). To collect the maximum amount of muscle activity, the probe was placed perpendicular (transverse) to the forearm within 30% to 50% of the length of the forearm from the elbow (Figure 5B). Moreover, to minimize the effect of transducer relocation on accuracy, data were collected at different transducer locations.

### 3.3. Experiment 1: Performance of Offline Classification

In the first experiment, an offline classification experiment was conducted in the able-bodied group to evaluate the potential of SMG as a novel HMI method. The accuracy of the classification method with different machine learning classification/regression algorithms including DTC, nearest neighbor regression (NNR), decision tree regression (DTR), KNN, SVR-L, SVR-P, SVM, and RF were compared after training and validation data were collected from 10 able-bodied people. In the final stage, for further evaluation of the developed model, nested and non-nested cross-validations were utilized.

#### Data Collection for Offline Testing

In the offline test, the able-bodied group were asked to sit comfortably on a chair and place their elbow on a pillow, with the palm facing upward. Before collecting data for training and validation, the position of the ultrasound transducer was first defined and fixed, making sure that key muscles, including the flexor digitorum superficialis (FDS), flexor pollicis longus (FPL), and flexor digitorum profundus (FDP), were covered by the transducer (Figure 5A). Each subject was then asked to perform one of nine different hand gestures, including rest, individual finger flexion (index, middle, ring, little, and thumb), fist, pinch, and key pinch, and hold it for 5 s. All nine hand gestures were repeated three times. To avoid fatigue and spasm in the muscles, there were 15 s of rest between each hand gesture. In the offline testing of the able-bodied group, in total, 11,625 images were collected, and 8350 of them were used for training, while 3275 images (384 × 400 pixels) were used for validation.

### 3.4. Experiment 2: Real-Time Functional Performance

To evaluate the functionality and performance of ProRuka, the developed controlling system and prosthetic hand were tested in real-time experiments with two amputees, using a variety of hand function test kits. In experiment 2, the B&B test as well as the TB&B test, which is a modified version of the B&B test, and Action Research Arm Test (ARAT) were utilized to evaluate the functional performance of the prosthesis in daily activities. Before the evaluation session, the two participants with transradial amputation were asked to attend two training sessions to improve their skills in controlling the robot as well as become familiar with the prosthetic hand and the process of the evaluation session.

Box and blocks test: Gross manual dexterity is often evaluated using a test called the B&B test [67]. The evaluation kit consisted of a box with two squared compartments which are separated by a partition (Figure S1). One of the compartments was filled with 150 wooden cubes (25 mm^3^), combining in such a way that the blocks may be found to rest in a wide variety of positions. The number of blocks that were moved over the barrier in the allotted time of 60 s was how the test was scored. The participants were free to carry the blocks in whatever order they wanted, provided that their fingers passed the partition between the two compartments before releasing the block into the desired location.

Targeted box and blocks test: The TB&B test was performed with 16 (for 4 × 4 TB&B Test [68]) and 9 (for 3 × 3 TB&B Test [69]) blocks. The TB&B Test is an upper-limb functional task designed to elicit ecologically meaningful activities such as movement initiation and the grasping, transporting, and controlled releasing of items. In addition to its use in assessing patients’ functional improvement after undergoing rehabilitation, this test may also be used as an outcome measure in clinical studies of upper-limb transradial prosthetic devices [70]. A standard grid was placed on both sides of the compartment, and the volunteers were asked to move each block to the other side of the compartment into its mirrored location. The box was turned upside down so that the outside area could be used for the assessment, which made it simpler to complete and also enabled the prosthetic hand to avoid colliding with the box’s walls (Figure S2).

Action Research Arm Test: The ARAT, which is extensively used to measure arm function, is one of the most prominent hand function evaluation kits. The testing kit consists of 19 different items to assess the different grasping types and arm movement (Figure S3). The whole assessment process takes approximately 10 min, scores are given based on the participants’ arm movement and functionality, and for each item, the score is rated between 0 (no movement) and 3 (normal movement) [71,72]. ARAT scores vary from 0 to 57, with 57 indicating higher performance. The final score indicates weak (less than 10), moderate (10–56), or excellent (57) hand function [73].

#### Data Collection for Real-Time Classification Testing

In the real-time classification experiment (experiment 2), to evaluate the whole SMG controlling system in the last session, different functional hand gestures including rest, pinch, key pinch, and cylindrical grip (fist) were classified as useful grasping types to help the participants use the robot in their ADLs. It is worth mentioning that out of the four available grips, the AI model was only trained with a cylindrical grip, since the robot was not able to perform other grips.

During the experiment for real-time classification, data were collected using one static and two different dynamic strategies, as the ultrasound image for each hand gesture varied due to hand movements while performing different tasks. In the static strategy, the same as in the previous experiment, ultrasound images from forearm muscles were collected while the participant’s hand was placed on the table with the palm upward (Figure 5C). In the first dynamic strategy, the participants were first asked to extend their hands and keep their palm in a supination position, then flip their hand without trying to move their wrist (flipping 90 degrees) while performing and holding one of the hand gestures (Figure S4). This activity was performed at a moderate speed and repeated three times for each hand gesture (rest, pinch, key grip, and fist). In the second dynamic strategy, the volunteers were asked to extend their elbow and then rotate their forearm 180 degrees three times while performing and holding one of the four hand gestures. They were instructed to perform the rotations at a moderate speed, defined as 1–2 s per complete rotation (180 degrees). This speed was monitored to ensure consistency across trials, with a target rotation speed of approximately 90 degrees per second. The amputee subjects were asked to repeat this process twice. The whole process for each hand gesture took 120 s, with a total of 480 s for the four hand gestures.

### 3.5. Results

#### 3.5.1. Offline Classification Results

**Figure 6:**
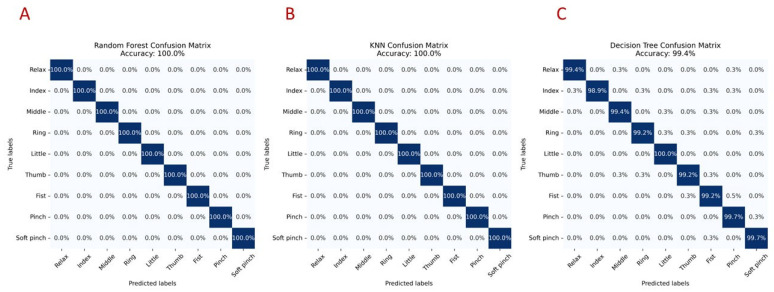
The results of the offline test: Offline test results for classifying nine different hand gestures in the 10 able-bodied participants group. VGG16 was used to extract features from the collected data, and these features were used to train an RF, KNN, or DTC model. The figure shows confusion matrices for hand gesture classification using the (**A**) Random Forest, (**B**) KNN, and (**C**) decision tree algorithms.

The offline classification results showed that the combining of a transfer learning model with one of the machine learning classification algorithms (KNN, RF, SVM, DTC) as well as regression methods, including nearest neighbor regression and decision tree regression, had the potential to classify the nine different hand gestures with an accuracy of more than 91% (Figure 6). However, training the model using SVR-L, SVR-P, and MLP showed significantly poorer performance in classifying the different hand gestures, with accuracies of 55.96%, 55.38, and 23%, respectively. Table 1 and Table 2 summarize the offline classification results obtained using various machine learning and regression algorithms and transfer learning approaches.

**Figure 7:**
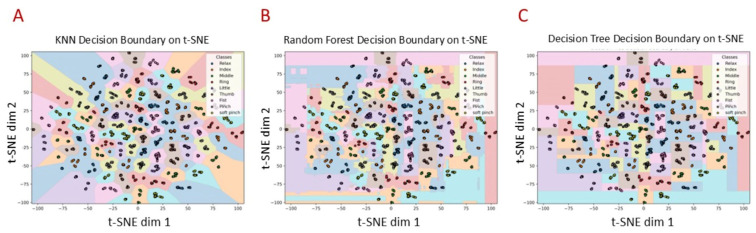
Two-dimensional t-SNE visualization: 2D t-SNE visualization of extracted features from transfer learning model with decision boundaries learned by (**A**) KNN, (**B**) RF, (**C**) DTC.

Figure 7 shows the 2D t-SNE visualization of the extracted features from the transfer learning model with decision boundaries learned by different classifiers. The t-SNE projection reveals well-formed clusters corresponding to each hand gesture class, indicating effective feature extraction. The KNN classifier produces smooth, well-separated decision regions, reflecting its strong ability to discriminate classes in this reduced space. In contrast, the Random Forest shows more fragmented boundaries due to its ensemble nature, while the Decision Tree Classifier creates axis-aligned, blocky partitions that result in less-smooth class separation. These visualizations provide intuitive insights into the classifiers’ differing strengths in handling complex feature distributions, with smoother boundaries suggesting better generalization and more fragmented or blocky boundaries indicating sensitivity to local patterns or abrupt transitions in the data.

To evaluate the performance and generalizability of our model, we applied a cross-validation (CV) scheme to the offline experiment. We tested the CV on models trained using the RF, DTC, or KNN machine learning algorithms, since these models showed the maximum accuracy in the offline test (100% accuracy). The statistical analysis of the cross-validation scores revealed a mean performance of approximately 99.8, indicating strong model effectiveness. The median score of 99.5 and mode of 99.5 further emphasize consistent performance across evaluations. With a range of 0.6, variance of 0.0712, and standard deviation of 0.2667, the scores exhibited minimal variability, suggesting the reliability of the results. The calculated margin of error for a 95% confidence level was ±0.197, leading to a confidence interval of [99.54, 99.94], which indicates that the true mean performance is likely to fall within this range. These findings collectively highlight the model’s robustness, with a reliable performance.

It is worth mentioning that more time was needed to train the models using SVR-L, SVR-P, decision tree regression, nearest neighbor regression, DTR, and SVM, while the RF and KNN models were the fastest in training using the collected datasets (around 205 s for the ten able-bodied volunteers, with 8350 images for training and 3275 images for validation).

#### 3.5.2. Real-Time Performance Results

Based on the offline test results, VGG16 was used to extract features and an RF machine learning algorithm was utilized to train the model (the accuracy of classifying the different hand gestures using this method was the highest). The two volunteers were invited to attend the experiment conducted to evaluate the functionality of the developed prosthesis. They were asked to complete the different hand function tests with the prosthesis in addition to their healthy hand to compare the results.

The final scores and results of the hand function test are summarized in Table 3. During the experiment, it was observed that a minimum of 120 s was needed to collect the training data for each hand gesture with an accuracy of 100%. It was also observed that the accuracy of classification was minimally reduced after transducer replacement due to putting on and taking the prosthesis. However, during data collection for training, data were collected at different transducer locations to minimize the effect of transducer relocation on accuracy.

The results of the B&B and TB&B tests demonstrated that the volunteers were able to pick up the blocks via pinching and successfully move them to the other side of the box without any misclassification during hand movements. Both of the participants were able to easily transfer around 13 blocks within 60 s without any prior training. However, the prosthetic hand exhibited limited fine dexterity, as evidenced by the relatively low number of blocks transferred compared to typical healthy hand performance. These limitations are likely attributable to the lack of sensory feedback and the rigid structure of the prosthetic hand, which reduce the user’s ability to modulate their grip force and adapt movements dynamically, thereby contributing to reduced efficiency and increased user fatigue during task execution.

The outcome of the ARAT demonstrates good performance in grasping and gripping different objects of different sizes, indicating reliable power and precision grips. Nonetheless, ProRuka struggled with fine motor tasks, such as picking up small objects, which require a delicate pinch gesture. Based on the scores earned by the volunteers, the prosthetic hand’s overall functional performance corresponds to that of a hand with moderate function. More specifically, individuals can perform basic tasks but may struggle with more complex or fine motor activities.

#### 3.5.3. Evaluating the Potential of Using a Silicone Pad Instead of Ultrasound Gel or a Gel Pad

In the experiment conducted to evaluate the potential of the silicone pad to be replaced with ultrasound gel in order to control the prosthesis using ultrasound imaging, we observed that a silicone pad could provide real-time images of the muscle with good image quality and that the captured data could be utilized to enable real-time control over the prosthetic hand. Moreover, it was also discovered that the sticky silicon pad did not only stop transducer relocation but also reduced the stress on the skin by dampening the transducer’s reaction force.

## 4. Discussion

SMG is a novel HMI method that allows users to control a prosthetic hand by capturing the residual muscles’ activities using ultrasound imaging. Figure 1 illustrates the SMG method as a new HMI technique for controlling prostheses with multiple degrees of freedom. In this study, the potential to control a prosthetic hand using SMG was evaluated. To classify different hand gestures, a combination of transfer learning models (including VGG16, VGG19, and InceptionResNetV2) and machine learning algorithms were utilized. And the results show that this new method has high potential to be utilized in the control of prosthetic hands.

The offline classification results showed that combining one of the transfer learnings with algorithms such as Random Forest, k-nearest neighbors, Decision Tree Classifier, Support Vector Machine, and regression methods yielded accuracies exceeding 91% for hand gesture recognition. Conversely, models based on support vector regression (linear and polynomial) and a multi-layer perceptron demonstrated substantially lower accuracy, between 23% and 56%. Cross-validation further validated the robustness of the top-performing models, with an average accuracy of approximately 99.8% and minimal variability across evaluations.

**Figure 8:**
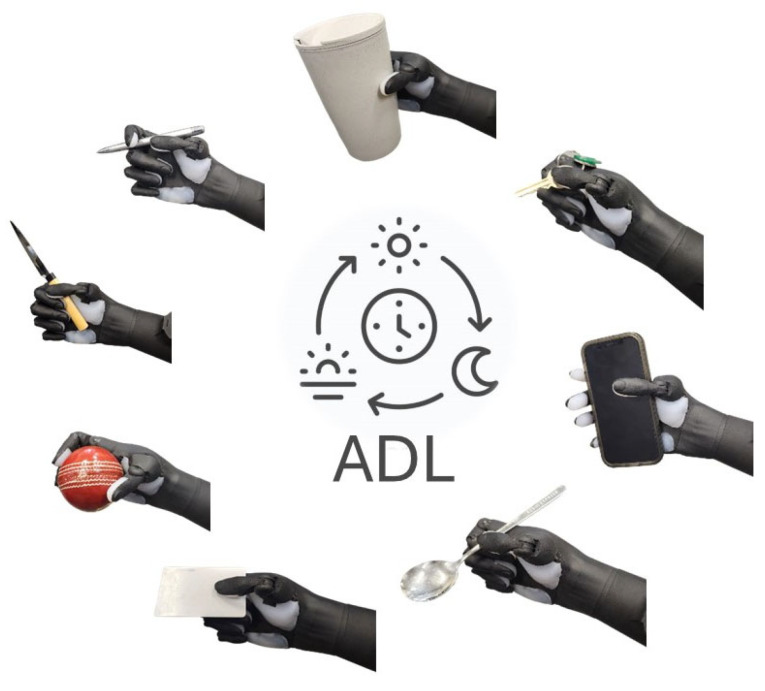
ProRuka in activities of daily living: Novel SMG system enabling multi-degree-of-freedom prosthetic hand to be used in daily activities.

In the functional evaluation test, we found that the volunteers who attended our study were able to control the prothesis and execute the different hand gestures needed for ADLs without any previous training. We found that collecting data from participants’ hands while they performed movements and rotated their wrists (see Figure S1) significantly reduced the misclassification errors during changes in arm position. This approach enhances the reliability of the control system, making it suitable for use in real-world settings beyond the laboratory. Moreover, the scores achieved by the two volunteers in the ARAT show that the developed SMG system to control the prosthetic hand has the potential to assist people with transradial hand amputation to perform different hand gestures needed for ADLs (Figure 8), and the scores also prove that the functionality of the prosthesis is as good as a hand with moderate hand function. The B&B and TB&B tests showed the functionality of the developed robot with this novel controlling system in regard to manipulating objects using pinching. Moreover, in the experiments, no misclassification during hand movements was observed when the volunteers wanted to transfer blocks (Supplementary Videos S1–S3).

During data collection and the testing of the SMG controlling system, we noticed that gel pads and ultrasonic gels increased the possibility of probe movement, which significantly lowered the precision and reliability of the SMG controlling system. In addition to this, the skin is in jeopardy due to the prolonged contact with moisture. Additionally, gels contaminate the environment in which the ultrasound probe is mounted. Several potential solutions to these problems have been proposed and evaluated by researchers in the last few years. For instance, Wang et al. recently created a bioadhesive ultrasound (BAUS) device that can provide pictures from organs for 48 h. To securely adhere an array of piezoelectric elements to the skin without ultrasound gel, they utilized a soft, tough, anti-drying, and bioadhesive hydrogel–elastomer hybrid couplant layer [74].

In this study, we proposed the utilization of a biocompatible sticky silicone pad as an alternative to ultrasound gel. It was discovered that a silicone pad has the potential to be used instead of ultrasound gel or gel pads, avoiding skin contact with moisture and thereby serious skin problems. We also observed that sticky silicone pads can not only help to capture images from muscles with good resolutions but also, by increasing the friction between the transducer and the skin, prevent the relocation of the transducer, resulting in a decrease in misclassification in real-time control. In addition, during the offline test, the accuracy of hand gesture classification in the able-bodied group was around 99% when ultrasound gel was used to collect the data. However, when the ultrasound gel was replaced with a silicone pad, the accuracy increased to 100%.

It is well known that muscles become fatigued under continuous contraction. The effect of this phenomenon for prothesis control using electromyography has been reported in previous studies [75,76]. The EMG signal magnitude increases during muscle fatigue, while the center frequency of the EMG reduces. Therefore, a change in frequency can be used to compensate the EMG magnitude, as used in Park and Meek’s study in 1993 [75]. In a recent study in 2019 [76], the data for training a model also included EMG signals collected under the fatigue situation; thus, the prediction model for providing prosthesis signals could work with both un-fatigued and fatigued muscle. Shi et al. (2007) demonstrated that SMG signals could also be used to evaluate muscle fatigue; i.e., muscle thickness increased during muscle fatigue [77]. This finding also indicated that muscle architectures change under the muscle fatigue status. In future studies related to SMG prosthesis control, it is important to include training images collected under the fatigued status when developing the model.

In addition, after the prosthesis is used for a period, the residual muscle of the amputee subjects may change as time goes by. For example, the residual muscle may become stronger, leading to morphological changes, after the user continuously uses the prothesis for a certain period. Under such situations, the originally trained model may not be able to achieve very high accuracy. We propose two possible solutions for future further investigations. Firstly, users can periodically collect new images of their forearm muscles and update their training dataset, retraining the controlling system. Alternatively, the AI model can be designed to automatically update its dataset by capturing new images while the user is using the prosthetic hand. Eventually the model can retrain itself with the updated dataset during the charging process of the prosthetic hand.

### Limitations and Future Works

In this study, we used a large number of images collected from each amputee’s hand to train the model for real-time evaluation. In order to put our focus more on the functionality of the prosthetic hand and the capabilities of the controlling system, we decided to spend more time evaluating the functionality of the hand by training the model with the essential and functional hand gestures. In future studies, the control of more complete sets of hand gestures can be evaluated.

Even though the volunteers in this study were able to complete the various tasks, they found it difficult to pick up small objects due to a lack of sensory feedback. In the hand function test, they tried to control the prothesis only by looking at the hand movements without sensing the location of each finger, making it difficult for them to exercise excellent control over the prosthesis. Moreover, to develop a cost-effective prosthetic hand, a minimum number of actuators and electronic items was used. However, in the hand function test, it was observed that it was difficult for the participants to perform some daily activities due to the lack of wrist rotation. They could pick up blocks, but they needed to move their entire body to grasp and hold a glass, especially when simulating the pouring of water from one glass to another. Furthermore, sometimes the participants complained about the prothesis blocking their view, making it difficult for them to see the objects that they wanted to pick up. In addition, based on the test results, it was observed that the prothesis could perform the pinch gesture, but it was difficult for the subjects to pick up small objects like coins, paper clips, ball bearings, etc., via pinching. In addition, to control the robot using the SMG technique, a wireless ultrasound transducer was mounted in the socket. The ultrasound device used in this study had the dimensions of 110 × 56 × 10 mm^3^ and weighed approximately 80 g. To accommodate this device in the socket, we were obligated to make the entire prosthesis slightly thicker than typical myoelectric prosthetics.

One of the main concerns regarding the SMG system is its long-term feasibility and power consumption. Although the SMG control system demonstrated promising accuracy, this performance can be influenced by changes in muscle structure due to fatigue or alterations in muscle tone over time. Additionally, ultrasound devices typically have higher power consumption compared to other sensing methods such as electromyography (EMG), which may limit their battery life and continuous use duration. These factors could impact the practicality and user comfort of the prosthesis during extended daily use, underscoring the need for the ongoing optimization of sensor design and improvements in energy efficiency.

In the future, different non-invasive methods for giving sensory feedback to amputees will be studied to not only increase the functionality of the hand but also decrease the occurrence of phantom pain in people with hand amputations [78,79,80,81]. Moreover, by increasing the DOF of the prosthesis and adding one more rotational joint in the thumb and one in the wrist, we will improve the dexterity, pinching, and wrist rotational movement of the prosthetic hand [82,83,84]. To remedy the limitations caused due to the rigidity of the prosthetic hand, in the future, a combination of rigid items and soft materials will be utilized to modify the prothesis and make it more like a human hand, with higher dexterity and flexibility [10,13,85,86,87]. Finally, different AI methods will be used to predict not only the intended hand gestures, but also the amount of intended finger flexion. This will provide proportional and natural control over prosthetic hands.

## 5. Patents

US patent, US application no. 18/305,4415, pub. No. US2024/0225861 A1 title: PROSTHETIC HAND DEVICE USING A WEARABLE ULTRASOUND MODULE AS A HUMAN MACHINE INTERFACE [88]

## Figures and Tables

**Table 1:** This table summarizes the performance of various machine learning algorithms using different transfer learning models in classifying nine different hand gestures. Accuracy is presented as a percentage, indicating the effectiveness of each method.

Machine Learning Algorithm	Transfer Learning Model	Accuracy
Random Forest (RF)	InceptionResNetV2	100%
K-Nearest Neighbors (KNN)	InceptionResNetV2	100%
Decision Tree Classifier (DCT)	InceptionResNetV2	100%
Support Vector Machine (SVM)	InceptionResNetV2	100%
Random Forest (RF)	VGG19	100%
K-Nearest Neighbors (KNN)	VGG19	100%
Decision Tree Classifier (DCT)	VGG19	100%
Support Vector Machine (SVM)	VGG19	100%
Random Forest (RF)	VGG16	100%
K-Nearest Neighbors (KNN)	VGG16	100%
Decision Tree Classifier (DCT)	VGG16	100%
Support Vector Machine (SVM)	VGG16	100%
Multi-Layer Perceptron (MLP)	VGG16	23%

**Table 2:** This table displays the performance of various regression algorithms using VGG16 for transfer learning in classifying nine different hand gestures. Accuracy is shown as a percentage, reflecting the effectiveness of each regression method.

Regression Algorithm	Accuracy
Neural Network Regression (NNR)	100%
Decision Tree Regression (DTR)	91.72%
Support Vector Regression (SVR-L)	55.96%
Support Vector Regression (SVR-P)	55.38%

**Table 3:** Result of hand function evaluation using B&B test, TB&B (4 × 4) test, TB&B (3 × 3) test, and ARAT. Both volunteers were right-handed and had left-hand transradial amputations.

Test	Hand	Result	
B&B		Number of blocks	
		A1	A2
	Left	12	8
	Right	45	47
TB&B (4 × 4)		Time (seconds)	
		A1	A2
	Left	86.66	136.79
	Right	31.31	21.23
TB&B (3 × 3)		Time (seconds)	
		A1	A2
	Left	41.40	67.18
	Right	17.00	12.28
ARAT		Score (total)	
		A1	A2
	Left	40	40
	Right	57	57

B&B: box and blocks; TB&B: targeted box and blocks; ARAT: Action Research Hand Test.

## Data Availability

All data and materials are available in the main text or the Supplementary Materials.

## Supplementary Materials

The following supporting information can be downloaded at: https://www.mdpi.com/article/10.3390/s25133968/s1. Figure S1: The box and blocks test kit; Figure S2: The targeted box and blocks test; Figure S3: The Action Research Arm Test kit; Figure S4: Flipping hand without moving wrist; Video S1: Assessing the functionality of the ProRuka using the box and blocks (B&B) test; Video S2: Evaluating the functionality of the ProRuka using the 4 × 4 targeted box and blocks (TB&B) test; Video S3: Assessment of ProRuka’s functionality using 3 × 3 TB&B test; Table S1: Comparison of mainstream prosthetic hand control technologies including SMG and EMG [57,58,59,60,61,62,63,64,65].

## Abbreviations

The following abbreviations are used in this manuscript:

HMI	Human–Machine Interface
SMG	Sonomyography
EMG	Electromyography
EEG	Electroencephalography
MIRA	Myoelectric Implantable Recording Array
MM	Magnetomicrometry
SDA	Subclass Discriminant Analysis
PCA	Principal Component Analysis
SVM	Support Vector Machine
BP-ANN	Backpropagation Artificial Neural Network
DOF	Degree of Freedom
B&B	Box and Blocks
TB&B	Targeted Box and Blocks
ARAT	Action Research Arm Test
CNN	Convolutional Neural Network
RF	Random Forest
KNN	K-Nearest Neighbors
DTC	Decision Tree Classifier
SVR	Support Vector Regression
NNR	Nearest Neighbor Regression
DTR	Decision Tree Regression
FDS	Flexor Digitorum Superficialis
FPL	Flexor Pollicis Longus
FDP	Flexor Digitorum Profundus
ADL	Activity of Daily Living
