# Supplementary material for: A Highly Efficient HMI Algorithm for Controlling a Multi-Degree-of-Freedom Prosthetic Hand Using Sonomyography
Source: Sensors (Basel). 2025 Jun 26;25(13):3968. doi: 10.3390/s25133968 (PMC12251556; doi:10.3390/s25133968)
Supplement: Supplementary file 1 [file sensors-25-03968-s001.zip › sensors-3615382-supplementary(1).pdf]

Table S1 provides a comparative analysis of the various mainstream technologies utilized in prosthetic hand control, focusing on key factors such as accuracy, latency, weight, and cost. By examining these parameters, we aim to highlight the strengths and weaknesses of each technology, including electromyography (EMG) and sonomyography (SMG). Accuracy is essential for ensuring the precise control of the prosthetic hand, while latency affects the responsiveness of the device. Weight is a significant consideration for user comfort and usability, particularly for extended wear, and cost plays a crucial role in accessibility for patients. This comprehensive overview enables a better understanding of how each technology measures up against the others, facilitating informed decision-making for developers and users in the field of prosthetic advancements.

Table S1: Comparison of mainstream prosthetic hand control technologies including SMG and EMG [56-64].

Technology	Typical accuracy	Latency	Device weight	Cost (USD)	Advantageous
SMG	91% – 97%	~150–250 ms	~70 – 400 g	$650 - $20,000 +	High spatial resolution; detects deep muscle deformation; less affected by muscle cross-talk; promising for multi-DOF control
EMG	74%-95%	~150–300 ms	9 – 100 g	$30–30000 +	Widely used; good accuracy and low latency; affected by electrode placement and muscle cross-talk; mature technology

We also included the following references related to the table in the main manuscript. We have highlighted these referneces for your refernece.

56. Maibam, P. C.; Pei, D.; Olikkal, P.; Vinjamuri, R. K.; Kakoty, N. M., Enhancing prosthetic hand control: A synergistic multi-channel electroencephalogram. *Wearable Technologies* **2024,** 5, e18.
57. Prakash, A.; Sharma, S.; Sharma, N., A compact-sized surface EMG sensor for myoelectric hand prosthesis. *Biomedical engineering letters* **2019,** 9, (4), 467-479.
58. Dunai, L.; Segui, V. I.; Tsurcanu, D.; Bostan, V., Prosthetic Hand Based on Human Hand Anatomy Controlled by Surface Electromyography and Artificial Neural Network. *Technologies* **2025**, 1-20.
59. Kalita, A. J.; Chanu, M. P.; Kakoty, N. M.; Vinjamuri, R. K.; Borah, S., Functional evaluation of a real-time EMG controlled prosthetic hand. *Wearable Technologies* **2025,** 6, e18.
60. Chen, Z.; Min, H.; Wang, D.; Xia, Z.; Sun, F.; Fang, B., A review of myoelectric control for prosthetic hand manipulation. *Biomimetics* **2023,** 8, (3), 328.
61. Li, W.; Shi, P.; Yu, H., Gesture recognition using surface electromyography and deep learning for prostheses hand: state-of-the-art, challenges, and future. *Frontiers in neuroscience* **2021,** 15, 621885.
62. Parajuli, N.; Sreenivasan, N.; Bifulco, P.; Cesarelli, M.; Savino, S.; Niola, V.; Esposito, D.; Hamilton, T. J.; Naik, G. R.; Gunawardana, U., Real-time EMG based pattern recognition control for hand prostheses: A review on existing methods, challenges and future implementation. *Sensors* **2019,** 19, (20), 4596.
63. Kinugasa, R.; Kubo, S., Development of consumer-friendly surface electromyography system for muscle fatigue detection. *Ieee Access* **2023,** 11, 6394-6403.
64. Nazari, V.; Zheng, Y.-P., Controlling upper limb prostheses using sonomyography (SMG): A review. *Sensors* **2023,** 23, (4), 1885.
