# Supplementary figures and images for: A Highly Efficient HMI Algorithm for Controlling a Multi-Degree-of-Freedom Prosthetic Hand Using Sonomyography
Source: Sensors (Basel). 2025 Jun 26;25(13):3968. doi: 10.3390/s25133968 (PMC12251556; doi:10.3390/s25133968)

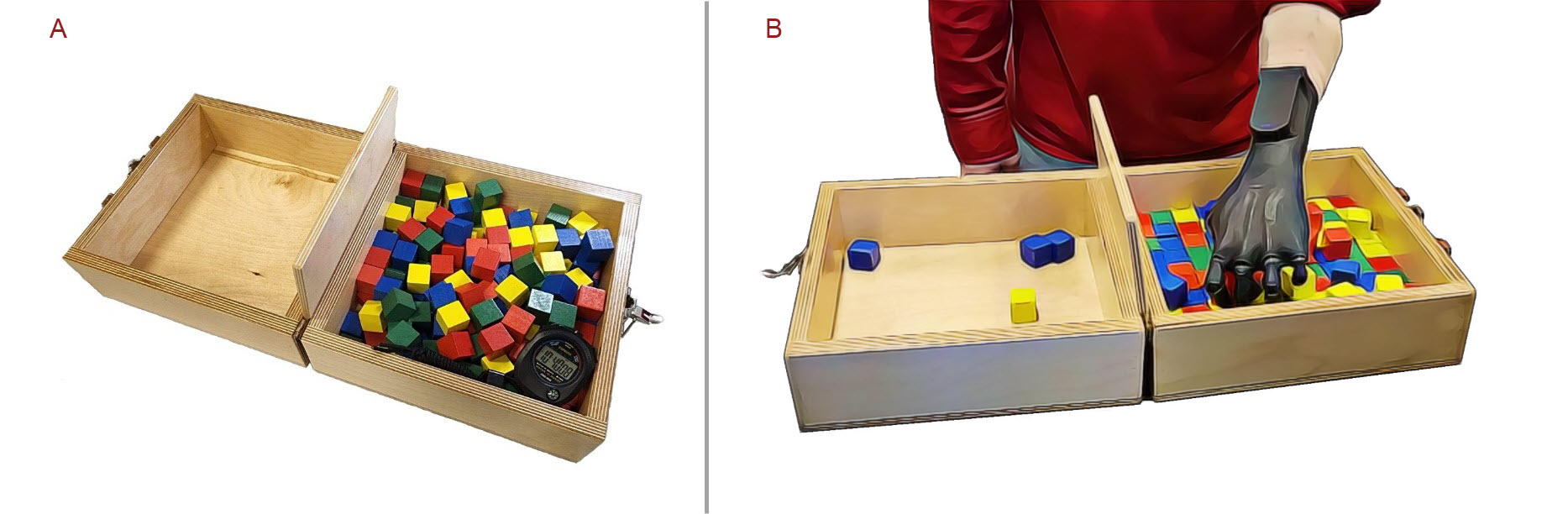

Supplement: Supplementary file 1 [file sensors-25-03968-s001.zip › S1.jpg]

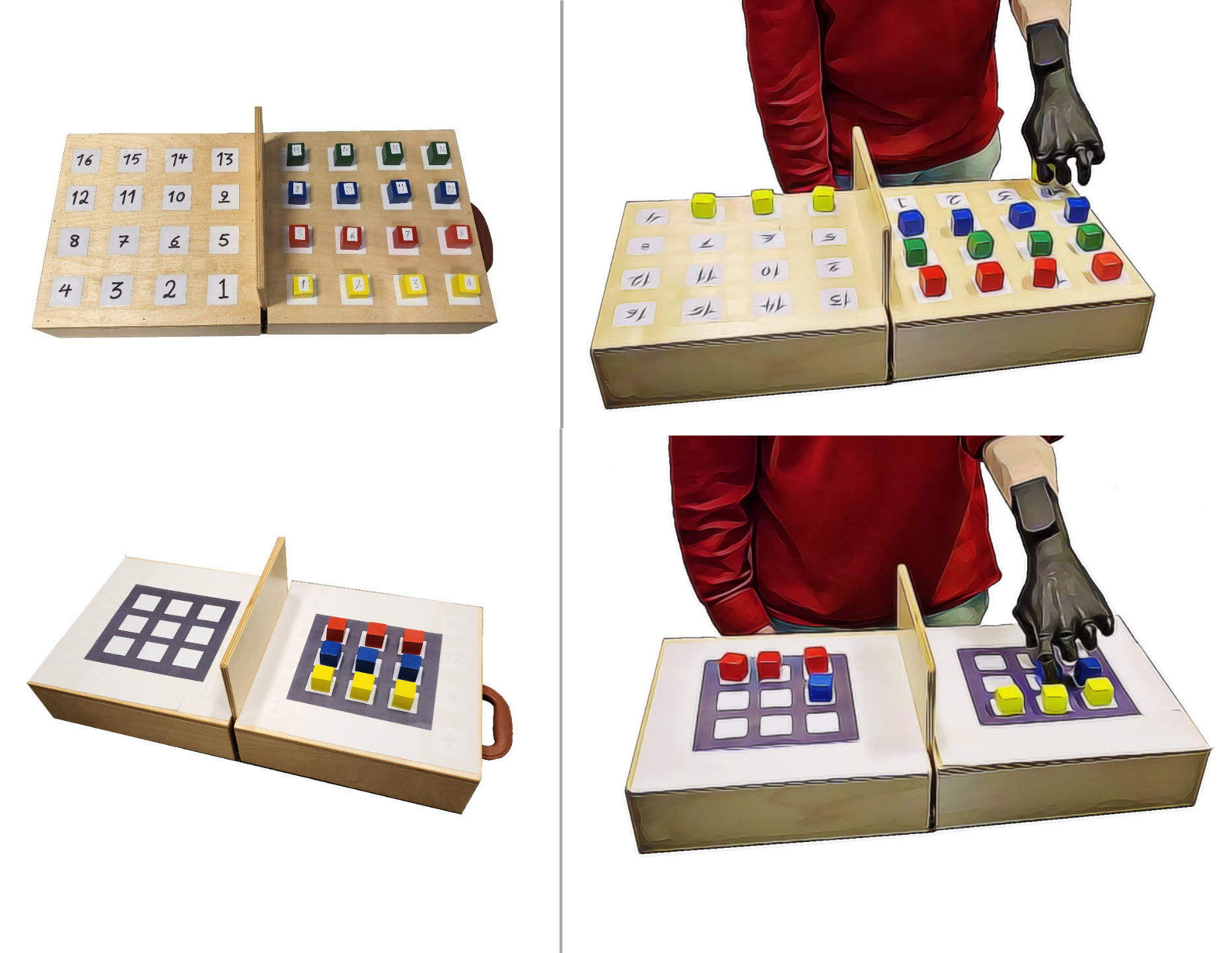

Supplement: Supplementary file 1 [file sensors-25-03968-s001.zip › S2.jpg]

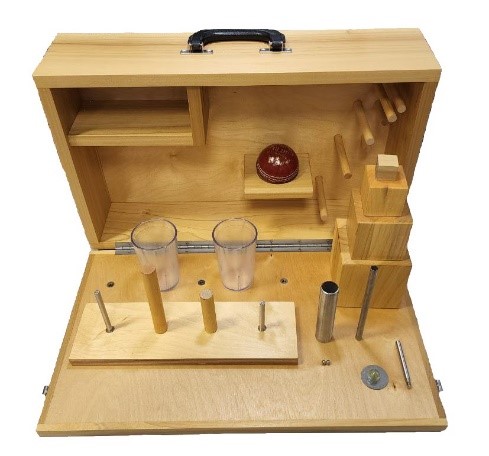

Supplement: Supplementary file 1 [file sensors-25-03968-s001.zip › S3.jpg]

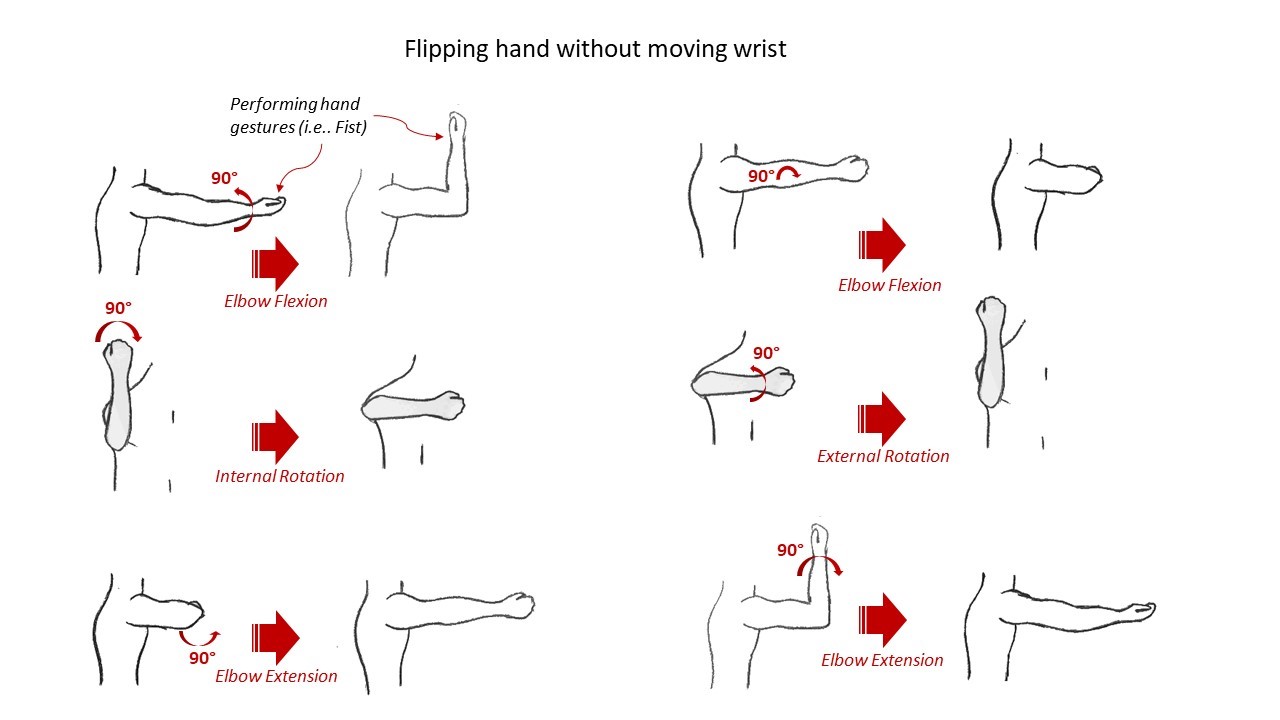

Supplement: Supplementary file 1 [file sensors-25-03968-s001.zip › S4.jpg]
